# The death of a myth: Females are not resistant to acute kidney injury

**DOI:** 10.1042/CS20257005

**Published:** 2025-08-13

**Authors:** Brian Soto Miranda, Carmen De Miguel

**Affiliations:** 1 Ponce Health Sciences University, Ponce, Puerto Rico, U.S.A.; 2 Section of Cardio-Renal Physiology and Medicine, Division of Nephrology, Department of Medicine, Heersink School of Medicine, University of Alabama at Birmingham, Birmingham, AL, U.S.A.

**Keywords:** AKI, females, kidney, subclinical injury

## Abstract

There is an important gap of knowledge regarding the mechanisms behind the greater prevalence of chronic kidney disease (CKD) in females compared with males. Most of the published reports suggest that females are protected from acute kidney injury (AKI) and from the AKI-to-CKD transition; however, in this issue of *Clinical Science*, Moronge et al. demonstrate that female rats present with subclinical markers of kidney damage post-ischemic reperfusion injury despite normalized levels of plasma creatinine. These studies underscore the potential for this AKI-induced subclinical injury to underlie the higher sensitivity of females to develop CKD later in life.

Historically, acute kidney injury (AKI) has been reported to be more prevalent among men than women, and consequently, most AKI studies have focused predominantly on the male population. However, women still comprise approximately 40% of AKI cases, demonstrating that a significant portion of them is affected by this condition [1] and highlighting the need to include them when studying AKI. While there is a shared belief that females are somewhat protected from AKI, different studies demonstrate that, globally, women have a higher overall age-adjusted prevalence of chronic kidney disease (CKD) compared with males [2–4]. Furthermore, studies also show that women with a history of AKI face an increased risk of adverse maternal and fetal outcomes during pregnancy [5,6]. It has also been suggested that this higher prevalence of CKD may be due to the presence of subclinical kidney injury that may predispose women to CKD; yet, the underlying mechanisms driving these outcomes remain poorly understood.

In this issue of *Clinical Science*, findings by Moronge et al. [7] emphasize the need to explore the impact of AKI on renal function in females and its long-term health implications. This study is the first to evaluate the persistence of subclinical renal injury in female rats following renal ischemia-reperfusion (IR), with the aim of uncovering the mechanisms contributing to adverse health outcomes later in life and applying this knowledge to women in the clinic. Figure 1 summarizes the findings of these studies.

**Figure 1:**
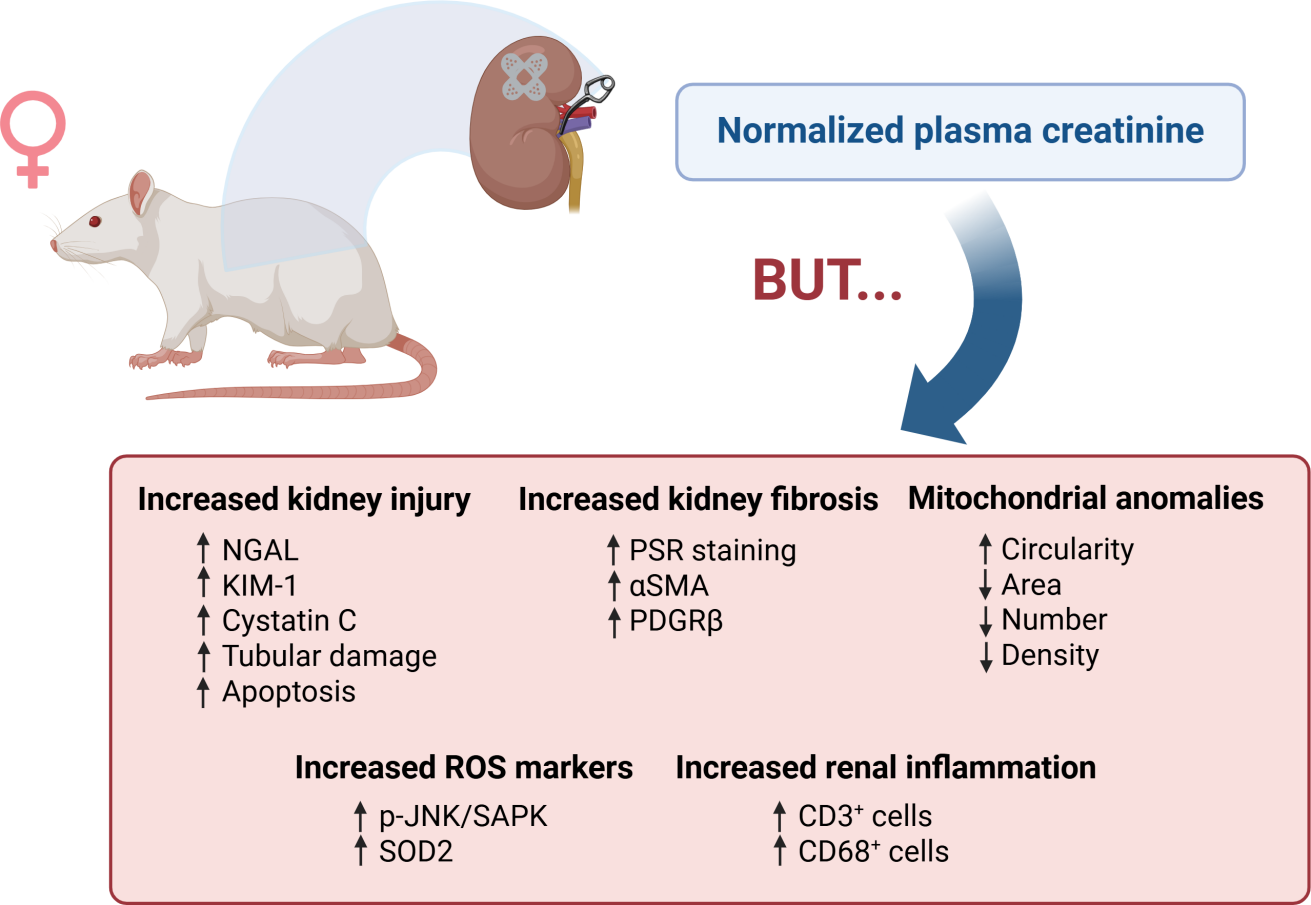
Subclinical renal injury exists in the female rat after ischemia-reperfusion (IR) injury. Thirty days after IR injury, female rats show normal levels of plasma creatinine; however, they also present with elevated markers of kidney damage, inflammation, and mitochondrial abnormalities. αSMA, α smooth muscle actin; CD3^+^ cells, T cells; CD68^+^ cells, macrophages; KIM-1, kidney injury marker-1; NGAL, neutrophil gelatinase-associated ligand; PDFGRβ, platelet-derived growth factor receptor β; p-JNK/SAPK, Jun-amino-terminal kinase/stress-activated protein kinase; PSR, picrosirius red. Figure created with BioRender.

The authors demonstrate persistent subclinical injury in young female rats following AKI, as evidenced by sustained elevations in renal injury markers neutrophil gelatinase-associated ligand (NGAL), kidney injury marker-1 (KIM-1), and cystatin C despite normalization of plasma creatinine levels. Their findings are consistent with previous studies by Dong et al., which reported similar results in male rats [8]; such observations had not previously been documented in females. The results by Moronge et al. carry important clinical implications, as they reveal the limitations of serum creatinine in assessing full renal recovery and underscore the need for implementing more sensitive biomarkers of kidney damage. Creatinine has low sensitivity and only begins to rise after approximately 50% of renal function has been lost [9], severely restricting its utility in detecting subclinical injury. Emerging biomarkers such as those evaluated in this study offer promise in identifying AKI at earlier stages and provide a tool for achieving a more accurate monitoring of recovery. In particular, a multicenter pooled analysis by Haase et al. examined a total of ten databases reporting the diagnostic and prognosis ability of NGAL in the context of AKI. They demonstrated that approximately 20% of critically ill patients exhibited an elevation in urinary or plasma NGAL levels without a corresponding rise in creatinine. This subgroup of patients experienced higher rates of adverse outcomes, including increased mortality [10]. These findings support the clinical value of incorporating subclinical renal injury markers like NGAL or KIM-1 into standard practice for monitoring and preventing the long-term sequelae of AKI.

Histological analysis in these studies also revealed mild renal fibrosis in female rats as early as 14 days post-AKI, accompanied by progressive tubular dilation, hyaline cast formation, and inflammatory cell infiltration beginning on day 3 and worsening by day 30. Those results suggest chronic and evolving renal damage, even in the absence of significant functional decline, as serum creatinine levels had normalized. Previous work in male rats had shown similar histological changes after IR, although often with earlier onset fibrosis [11], suggesting potential sex differences in the fibrogenic response to AKI. Clinically, this is significant because a seemingly resolved episode of AKI in women may conceal ongoing structural damage. In human studies, women have often been underrepresented in renal biopsy series following AKI, leaving a gap in our understanding of fibrosis progression in female patients. The results by Moronge et al. help bridge that gap and reinforce the importance of sex-specific strategies for post-AKI monitoring, especially considering that renal fibrosis is a key driver of CKD progression [12].

Mitochondrial integrity, a cornerstone of renal tubular health, was also markedly compromised in female rats post-AKI. Reduction in mitochondrial number, increased circularity, smaller area, and degeneration of mitochondrial shapes are consistent with mitochondrial fragmentation, which is a key contributor to tubular cell injury and apoptosis [13]. Similar changes following IR in male rats were reported by Lan et al., suggesting that renal mitochondria are affected similarly in both sexes [14]. Changes in mitochondria ultrastructure may underlie the sustained histopathological and biochemical indicators of renal injury. Clinically, mitochondrial health may serve not only as a therapeutic target but also as a potential biomarker of renal recovery. Based on the outcomes from these studies, therapies aimed at preserving or restoring mitochondrial function, such as mitoprotective agents or targeted antioxidants, may be particularly beneficial in female patients, who are often perceived to be at lower risk and therefore under-monitored after AKI.

The authors also revealed an imbalance between reactive oxygen species (ROS) generation and detoxification in female rats. These two processes are involved in promoting cellular injury in the kidney and progression to CKD [15]. While superoxide dismutase (SOD) activity was unchanged 30 days post-AKI, other groups reported that genetic deletion of SOD2 induces a more severe pathology regardless of total SOD activity [16], suggesting that low levels of SOD2 could be directly contributing to the maladaptive recovery from AKI. Furthermore, persistent inflammatory cell infiltration in the kidney also underlines a chronic inflammatory state in females that may further drive fibrosis. Clinically, this is highly relevant as ongoing oxidative stress and inflammation after AKI are linked to increased risk of CKD progression [17]. In addition, the exaggerated cell death evidenced in female rats post-AKI ‘recovery’ further contributes to the subclinical deterioration of renal function. Clinical interventions that modulate ROS activity and inflammation could potentially minimize apoptosis and protect female patients from long-term complications post-AKI [18].

This research highlights that AKI studies should prioritize a comparative analysis including both sexes to strengthen any conclusions regarding biological variability in response to injury. Such comparative analysis is essential for delineating sex-specific mechanisms of renal injury, repair, and long-term sequelae. Additionally, extending the observational period beyond 30 days could provide insight into whether subclinical injury resolves or progresses to overt CKD. Prolonged follow-up could also reveal patterns of recovery not captured within shorter time frames. Another area of interest is hormonal regulation of renal recovery, as estrogens are known to influence both mitochondrial function and inflammatory pathways, resulting in a complex interplay that may drive sex-specific outcomes in recovery from AKI [19]. Future studies should also include direct measurements of ROS levels to determine whether oxidative stress plays a main role in the persistence of subclinical renal injury in females. Future investigations should also analyze the localization of fibrosis within the kidney and if the deposition of collagen differs between the kidney cortex and medulla or between the sexes. Finally, and given the observed structural alterations in mitochondria, the therapeutic potential of mitochondrial-targeted interventions should be explored as they may offer novel strategies for halting or reversing injury at the subcellular level – particularly in the female population. Collectively, these possible future directions would expand our understanding of sex differences in AKI and support the development of more personalized post-AKI clinical care.

The results by Moronge et al. have key clinical translational value as they demonstrate that, contrary to what is often reported, females are not totally resistant to experimental AKI. The authors clearly show that a history of AKI in females leaves important sequelae in the kidney that may explain the greater prevalence of CKD observed in female populations later in life. Their findings have the great potential of changing clinical practice by using new markers in the diagnosis and treatment of AKI in females and underscore the need to not depend solely on values of plasma creatinine in these patients.

**Clinical Perspective**

- Females undergo important acute kidney injury (AKI)-induced changes in the kidney despite presenting with normalized values of plasma creatinine.
- New markers of kidney damage such as neutrophil gelatinase-associated ligand and kidney injury marker-1 should be routinely added in the clinic for the diagnosis of AKI in female patients.

## Open Access

This article has been published open access under our Subscribe to Open programme, made possible through the support of our subscribing institutions, learn more here: https://portlandpress.com/pages/open_access_options_and_prices#conditional

## Abbreviations

AKI: acute kidney injury
CKD: chronic kidney disease
IR: ischemia-reperfusion
KIM-1: kidney injury marker-1
NGAL: neutrophil gelatinase-associated ligand
ROS: reactive oxygen species
SOD: superoxide dismutase
